# Automated computer-assisted detection system for cerebral aneurysms in time-of-flight magnetic resonance angiography using fully convolutional network

**DOI:** 10.1186/s12938-020-00770-7

**Published:** 2020-05-29

**Authors:** Geng Chen, Xia Wei, Huang Lei, Yang Liqin, Li Yuxin, Dai Yakang, Geng Daoying

**Affiliations:** 1 Academy for Engineering and Technology, Fudan University, 20 Handan Road, Shanghai, 200433 China; 2 Suzhou Institute of Biomedical Engineering and Technology, Chinese Academy of Sciences, 88 Keling Road, Suzhou, 215163 China; 3 Department of Radiology, Huashan Hospital, Fudan University, 12 Wulumuqi Middle Road, Shanghai, 200040 China

**Keywords:** Cerebral aneurysm, TOF-MRA, Fully convolutional network, Computer-assisted detection

## Abstract

**Background:**

As the rupture of cerebral aneurysm may lead to fatal results, early detection of unruptured aneurysms may save lives. At present, the contrast-unenhanced time-of-flight magnetic resonance angiography is one of the most commonly used methods for screening aneurysms. The computer-assisted detection system for cerebral aneurysms can help clinicians improve the accuracy of aneurysm diagnosis. As fully convolutional network could classify the image pixel-wise, its three-dimensional implementation is highly suitable for the classification of the vascular structure. However, because the volume of blood vessels in the image is relatively small, 3D convolutional neural network does not work well for blood vessels.

**Results:**

The presented study developed a computer-assisted detection system for cerebral aneurysms in the contrast-unenhanced time-of-flight magnetic resonance angiography image. The system first extracts the volume of interest with a fully automatic vessel segmentation algorithm, then uses 3D-UNet-based fully convolutional network to detect the aneurysm areas. A total of 131 magnetic resonance angiography image data are used in this study, among which 76 are training sets, 20 are internal test sets and 35 are external test sets. The presented system obtained 94.4% sensitivity in the fivefold cross-validation of the internal test sets and obtained 82.9% sensitivity with 0.86 false positive/case in the detection of the external test sets.

**Conclusions:**

The proposed computer-assisted detection system can automatically detect the suspected aneurysm areas in contrast-unenhanced time-of-flight magnetic resonance angiography images. It can be used for aneurysm screening in the daily physical examination.

## Background

Among people without comorbidity, with an average age of 50 years, the prevalence of unruptured intracranial aneurysms is about 3.2% in a population without comorbidity [1]. Though it has a strong latency, some aneurysms may show no symptoms for years or even decades, the rupture of one aneurysm may lead to serious neurological sequelae and may be fatal. Under such circumstances, the prediction of when an aneurysm will rupture becomes very important. Hence, an automated detection system for cerebral aneurysms may help clinicians in the earlier and more accurate diagnosis of aneurysms. TOF-MRA as a non-invasive imaging technique shows promising diagnostic accuracy compared with DSA, which is the gold standard diagnostic method for aneurysm [2]. Therefore, TOF-MRA is currently one of the most commonly used methods for screening aneurysms, of which 3.0 T is the most popular [3].

Deep neural networks have been used to detect cerebral aneurysms since 2017 [4–9]. Up until now, several methods have been proposed in this field [4–17]. Nakao et al. [6] detected 94.2% (98/104) of aneurysms with 2.90 FPs/case, with sensitivity of 70.0% at 0.26 FPs/case. Ueda et al. [8] obtained 91% sensitivity at 6.60 FPs/case. Hanaoka et al. [10] obtained 80.0% sensitivity at 3.00 FPs/case. However, the proposed works only use 2D CNN networks or hand-engineered features. Moreover, because fully convolutional network (FCN) has greatly improved the state-of-art in image segmentation, it also plays an important role in the detection of lesions in medical imaging [18–20].

On the other side, computer-assisted detection (CAD) system is not only one method that detects lesions in the medical images, but it is also an end-to-end system composed of multiple algorithms with multiple steps. The purpose of the CAD system is to enable doctors to achieve faster and more accurate detection of lesions with the aid of computers without the need for excessive engineering knowledge.

In this paper, we developed a CAD system for cerebral aneurysms in TOF-MRA, using this system, the clinicians could get (1) a three-dimensional mesh model of intracranial artery, which could be used for hemodynamic analysis, and (2) the suspected areas of aneurysms, which were detected using an FCN-based network. The whole process is fully automated, clinicians only need to select the image data and then check if the area marked by the system is an aneurysm.

## Results

In this study, we used sensitivity and false-positive rates as indicators to evaluate the proposed method. All the aneurysms were considered positive. As the result of the system was a spherical area, if more than 30% of the aneurysm was in this spherical area, then this spherical area was considered a true-positive case, otherwise false-positive case.

We split the patients into three sub-datasets: a training dataset, an internal test dataset, and an external test dataset set. The number of cases in the three datasets was 76, 20, and 35, respectively. Using the internal test dataset, we do a fivefold cross-validation of the model, and the sensitivity is 94.41% ± 1.05%. Then we test the model in the external test dataset, which is not used in the training of the model. The characteristics of the external test dataset are shown in Table 1.

**Table 1:** The detailed characteristics of external test dataset

Characteristics	External test dataset
No. of patients	35
No. of male patients	13
No. of female patients	22
Mean age (year)	57 ± 14
Male patients (year)	59 ± 10
Female patients (year)	55 ± 15
Hypertension patients	18
No. of aneurysms	35
Mean size of aneurysms	6.48 ± 4.00
Size of aneurysms	
< 3.0	2
3.0–4.9	11
5.0–9.9	17
≥ 10.0	5
Location of aneurysms	
Internal carotid artery area	19
Middle cerebral artery area	5
Anterior cerebral artery area	8
Posterior cerebral artery area	3
Basilar artery area	0
Vertebral artery area	0

**Fig. 1:**
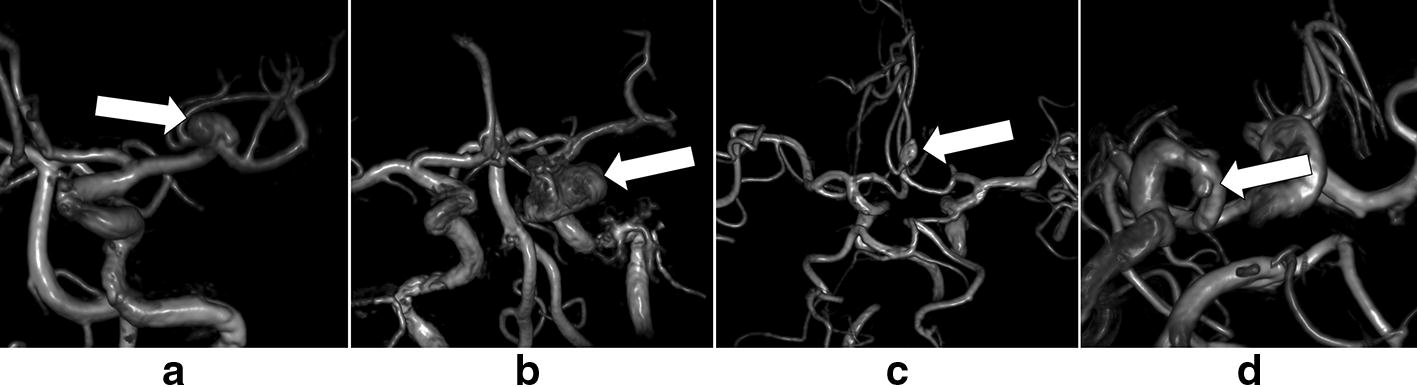
Examples of volume-rendered images. **a**, **b** The detected aneurysms by the proposed system. **c**, **d** The undetected aneurysms

The external test dataset is acquired with the same factors like the training dataset and internal test dataset. The characteristics of the external test dataset are shown in Table 1. Among the patients in the external test sets (35 patients totally, age ranges from 17 to 76), 22 are female (age range 17–76 years; mean age, 55 ± 15) and 13 are male (age range 42–75 years; mean age, 59 ± 10). Among these patients, 42% are over 60 years old, and 51.4% have hypertension. The max diameter of aneurysms ranges from 2.00 to 23.10 mm, and 40% of which are under 5 mm. The distribution of aneurysms covers the internal carotid artery area, middle cerebral artery area, anterior cerebral artery area, posterior cerebral artery area, but no basilar artery area and vertebral artery area. The aneurysms’ average size is 6.86 mm in the internal carotid artery area, 5.95 mm in the anterior cerebral artery area, 7.22 mm in the middle cerebral artery area and 4.28 mm in the posterior cerebral artery area, respectively. In the above areas, the largest aneurysm is located in the internal carotid artery area (Fig. 1).

**Fig. 2:**
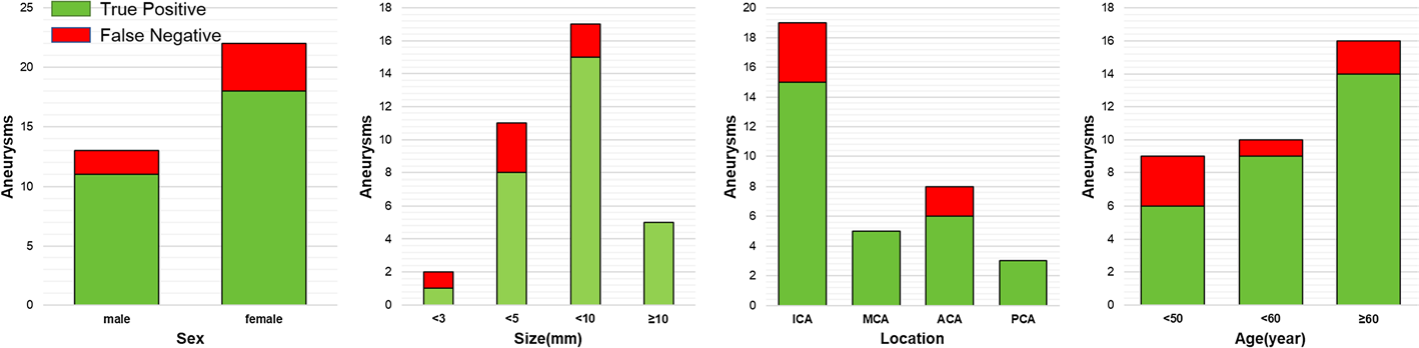
Subgroup analysis of sensitivity

Overall, 36 aneurysms were acquired in the external test dataset, annotated by two radiologists with 3 years’ experience, their annotations matched in these 35 cases and double-checked by one radiologist with 10 years’ experience. Using our CAD system to detect the aneurysms in the dataset, as we tune the threshold which defines whether a voxel is aneurysm or vessel, we find that the true-positive cases remain the same when the threshold is above 0. Our CAD system detected 82.9% of all the annotated aneurysms, with 0.86 false positive/case. Among the 6 undetected cases, 3 are female and 3 are male, age ranges from 35 to 69, 3 have hypertension, the distribution covers anterior cerebral artery, middle cerebral artery area, and internal carotid artery area, and the max diameter ranges from 2.60 to 5.67 mm. Our environment is CPU: Intel Core i9-9900K, RAM: 32 GB, GPU: NVIDIA GeForce RTX 2080Ti, Win10 professional, Tensorflow 1.14.0, Keras 2.0.8. In our environment, it took an average of 56 s to process one case of data and detect all possible aneurysms in the data (Fig. 2).

## Discussion and conclusion

In our study, TOF-MRA source images were obtained at 3.0 T with the same imaging parameters. The composition of age, gender and size are fully random, in line with daily conditions, which made the system more generalizable by minimizing over-fitting. The system detects 94.41% (mean of fivefold) and 82.9% (29 of 35) of aneurysms in the internal test dataset and external test datasets, respectively. By analyzing the result of the external test dataset, our CAD system performs the same in different gender subgroups and different age subgroups. The max diameter of the aneurysm has a significant effect on detection performance. The system detects 100% aneurysms with the max diameter larger than 10 mm, both in the internal test dataset and external dataset, and perform better in the detection of 5–10 mm objects than 3–5 mm ones. Since there are only 2 aneurysms smaller than 3 mm, the result cannot prove the performance on this size. The system detects 83.3% (15 of 18) of aneurysms in hypertension patients, which is basically the same as that for patients without hypertension. The proposed system detects all the aneurysms in the MCA and PCA areas and performs the same in the ICA and ACA areas. Through the results, it can be seen that the proposed system performs relatively well in various types of data. The three-dimensional network can make full use of the three-dimensional features of the data as a basis for judgment, and is, therefore, suitable for blood vessel data, especially for the judgment of similar structures such as vessel bifurcation, vascular angle, and vascular tumor. However, the problem with the three-dimensional network is that it requires more training data and is highly sensitive to noise. Direct use of the original data may result in incorrect classification results due to the complexity of the background. In the aneurysm CAD system proposed here, all the intracranial arterial regions containing the inside of the aneurysm were first extracted, and then a full convolutional network-based method was used to automatically detect the aneurysm, and the sensitivity was 82.9%. With the potential for practical applications, it is foreseeable that increasing the number and type of input data will significantly improve the performance and generalization capabilities of the system. Besides, introducing attention mechanisms or changing the network structure (e.g., DeepLabV3 [21]), better results may also achieved. We believe the methods above can improve our system and get better performance, and we will continue to work for that.

## Methods

### Materials

The ethics board of our institution comprehensively reviewed and approved the protocol of this study. Two of the authors of this paper (H.L. and L.Y.) are radiologists with 5 years and 10 years of work experience, respectively. They diagnosed all the aneurysms in this study, with the DSA as ground truth.

### Patients

A total of 131 patients (all have unruptured cystic aneurysm) underwent contrast-unenhanced 3D TOF-MRA. Patients were selected randomly from outpatient and physical examinations, with a period from 2016.03 to 2017.11. The aneurysms of the patients in this experiment were detected because the patients had symptoms such as headache, or were accidentally found due to other reasons. And all the sets were annotated by drawing the whole aneurysm areas. Then the patients were divided into three datasets: Training dataset, Internal Test dataset, and External Test dataset. Among the patients in training dataset and internal test dataset, 65 were female and 31 were male, age ranges from 28 to 86. Among these patients, 37.5% were over 60 years old. The max diameter of aneurysms ranges from 1.39 to 21.00 mm, and 38.5% of which were under 5 mm. The distribution of aneurysms covered the internal carotid artery area, middle cerebral artery area, anterior cerebral artery area, posterior cerebral artery area, basilar artery area, and vertebral artery area (Table 2). In the training dataset, there were 80 aneurysms (4 patients had double cases, and 72 patients had single case). In the internal test dataset, there were 25 aneurysms (4 patients had double cases, 1 patient had triple cases, and 14 patients had single cases). The aneurysms’ average size was 6.60 mm in the internal carotid artery area, 7.01 mm in the anterior cerebral artery area, 8.24 mm in the middle cerebral artery area and 6.42 mm in the basilar artery area, respectively. In the above areas, the largest aneurysm was located in the middle cerebral artery area.

**Table 2:** Detailed characteristics of training dataset and internal test dataset

Characteristics	Training dataset	Internal test dataset
No. of examinations	76	20
No. of male patients	24	7
No. of female patients	52	13
Mean age (year)	56 ± 11	56 ± 10
Male patients	56 ± 10	56 ± 10
Female patients	58 ± 13	56 ± 10
Hypertension patients	39	9
No. of aneurysms	80	26
Mean size of aneurysms	6.86 ± 4.23	6.30 ± 3.56
Size of aneurysms		
< 3.0	10	3
3.0–4.9	23	6
5.0–9.9	30	15
≥ 10.0	17	2
Location of aneurysms		
Internal carotid artery area	40	14
Middle cerebral artery area	13	9
Anterior cerebral artery area	11	2
Posterior cerebral artery area	13	0
Basilar artery area	2	1
Vertebral artery area	1	0

### Datasets

**Fig. 3:**
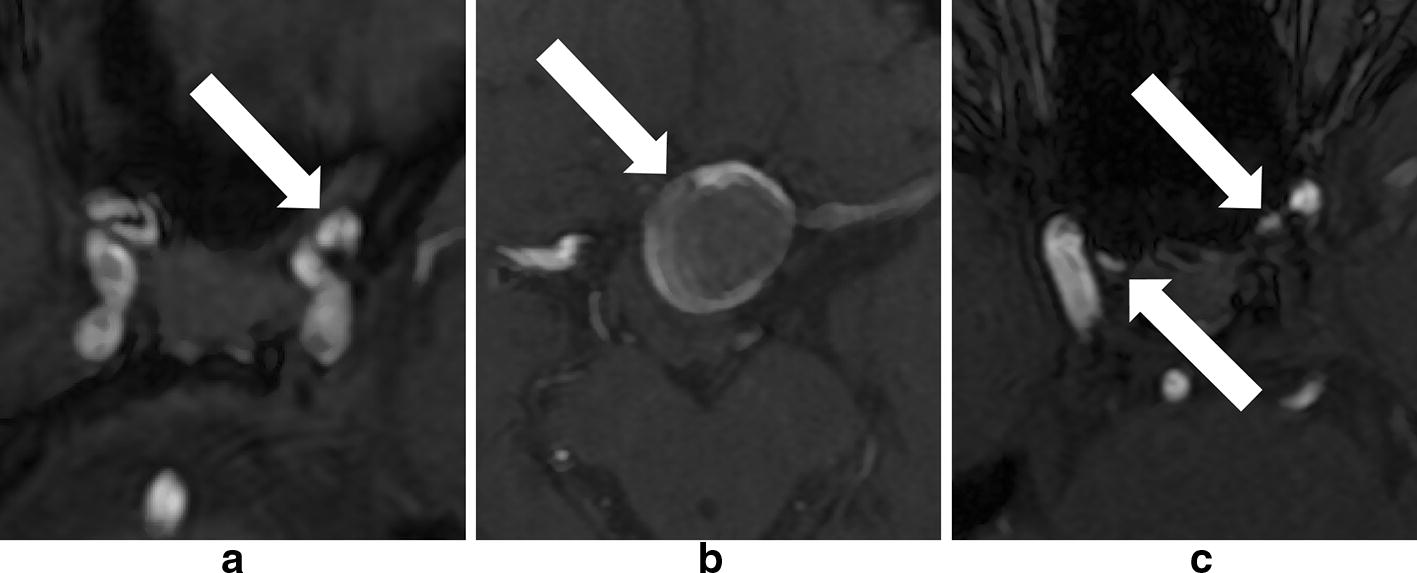
Aneurysms in our dataset **a** 4 mm single, **b** 22.3 mm single, **c** double aneurysms

In this study, TOF-MRA source images were used to develop the algorithms. All angiography examinations were performed with a 3.0-T system (GE Discovery MR750), with the same imaging factors (repetition time/echo time, 25 ms/5.7 ms; flip angle, 20°; field of view, 220 mm; section thickness, 1.2 mm; acquisition matrix, 320*256,reconstructed to 1024*1024; acquisition time, 2 min 14 s). A total of 131 TOF-MRA image sets were collected, of which 76 were training dataset, since we used a deep neural network-based algorithm to detect the aneurysms, 76 image sets were not enough to achieve sufficient classification performance for the network model. We augmented the image sets with flipping (by transverse section), discrete Gaussian noise filter (variance: 4.0, max kernel width: 32 pixels), and histogram equalization filter in turn, and finally got 608 image sets for training. The internal test dataset was also augmented in the same way and got 160 image sets. Of course, we also resampled all the image sets to isotropic and cropped them, so the no-content edges would not affect the training (Fig. 3).

### Development of the CAD system

**Fig. 4:**
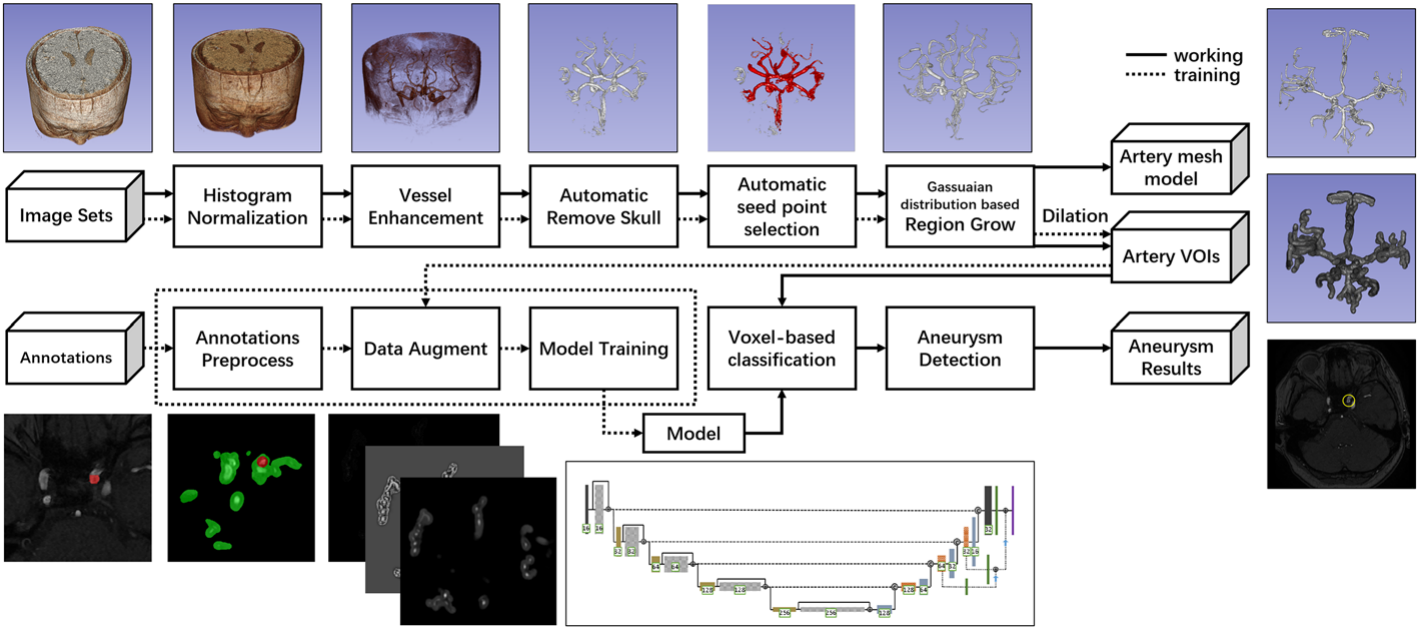
Workflow of the proposed CAD system for cerebral aneurysms

In our system, we designed two main steps: first, automatic segmentation of the artery vascular voxels; second, aneurysm detection based on deep neural networks. And there were two pipelines, one for the training of the neural network model, as the preparation of the system, the other for the actual detection of the aneurysms in the real data and show the results to users. So the clinicians only need to input DICOM image sets and then the system would show where the aneurysms were with a high likelihood (Fig. 4).

#### Step one: segmentation

The input was DICOM datasets, which is in the form of a volume. In view of the many three-dimensional features of cerebral arteries, our method processed the image data as volume from start to finish. In the segmentation step, the input image data were pre-processed using N3 bias field correction and histogram normalization. Then the vessel region was enhanced using a sigmoid filter, the total transform is given by the formula below, with *α* = 400 and *β* = 600:

$$f\left( x \right) = \left( {{\text{Max}} - {\text{Min}}} \right) \cdot \frac{1}{{\left( {1 + e^{{ - \frac{x - \beta }{\alpha }}} } \right)}} + {\text{Min}} .$$

**Fig. 5:**
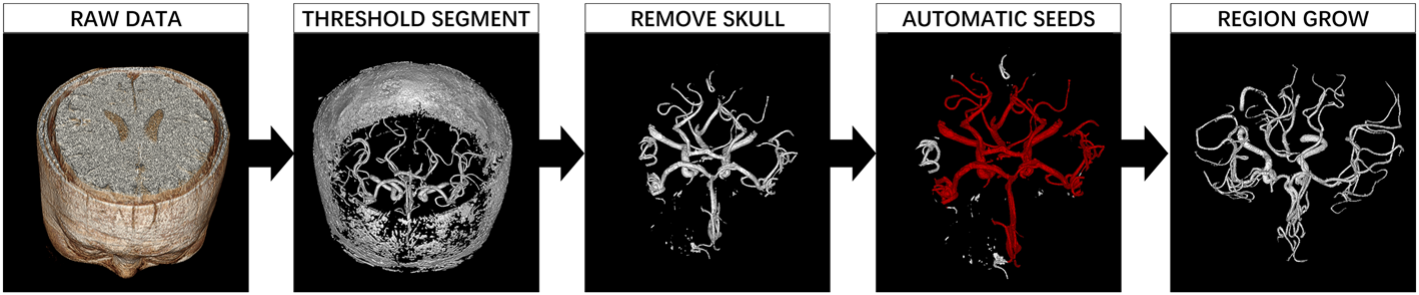
Workflow of the segmentation in the proposed CAD system

Seed points on skull were selected automatically based on the bounding-box method. This method used a cube to wrap the skull from the outside and shrank it. When the faces of the cube came into contact with the skull, the contact points were used as the seed points. The auto-threshold region was allowed to grow from the seed points and smooth the results, then the skull region voxels were obtained. The lower threshold of the region-growing method was 30% of the maximum intensity, and upper threshold was the maximum intensity. These voxels were cut off from the pre-processed data and the skull was removed. Since the high signal area in TOF-MRA data was mainly skull and vessels, artery blood vessel accounted for a large part of the left high signal objects. We binarized the data based on the intensity region of the vessel, set all voxels greater than the background density value to 1. Then we performed connected domain statistics, arranged connected domains according to the number of voxels it contains, and selected seed points from the 5 connected domains that are ranked first. In most instances, the selected seed area was the main branch of artery vessels. Since the intensity of the vessels in TOF-MRA data followed the Gaussian distribution [22], the upper and lower thresholds of the region-growing method were decided automatically as μ + σ and μ − σ (μ stands for the estimated mean and σ stands for the standard deviation). With the automatic region growth, vessel voxels were segmented from the TOF-MRA data, reconstructed the surface of the vessel using the marching cubes method, then the surface mesh model of artery vessels was obtained. The mesh model could be used in the hemodynamic analysis since it was the inner surface of vessels. The target of the CAD system was the detection of aneurysms; to do the detection, the vessel voxels were dilated using radius 10 sphere kernel, and the vessel area and its neighbor area were the input of step two. The auto-segmented vessel area output of step one covered 100% of the labeled aneurysms in our dataset (Fig. 5).

#### Step two: detection

In this step, we used a deep neural network to detect the aneurysms in the image. Since the blood vessel was a continuous structure in three dimensions, and usually had a long radial length, traditional 2D CNNs could not be applied on the important 3D structure features, which were widely used in clinical diagnosis. Fully convolutional networks (FCNs), such as U-Net, made full use of every pixel in the image and brought semantic segmentation to a practical level. In this paper, we chose an improved 3D-UNet [23] as our detection method. The structure of this network was below. Inspired by U-Net, this network could process 3D input blocks of 128*128*128 voxels, and also comprised a context aggregation pathway as U-Net. Besides, they employed deep supervision to the network by injecting gradient signals.

To train our model, we augmented the 96 image sets to 768, using flipping (by transverse section), histogram normalization, discrete Gaussian noise filter (variance: 4.0, max kernel width: 32 pixels, sequentially. The image sets were then cropped and resampled to 128*128*128. The annotations were dilated based on the center of the labeled area, all annotations were dilated to a sphere with the same radius. The way to choose the radius was above 3 voxels under 128*128*128, which was above 3% of the length of axis. After the dilation, the annotations had two labeled objects, the vessel area as background, and aneurysms as foreground. Then 76 of the 96 image sets were selected as training datasets and put into the network for training. The initial factors were: batch size = 1, initial learning rate = 5e−4, optimization function was Adam, the weights were initialized using the default initializer (glorot_uniform) of Keras. After about 200 epochs the learning process got an early stop; it costs 10 h in our environment.

**Fig. 6:**
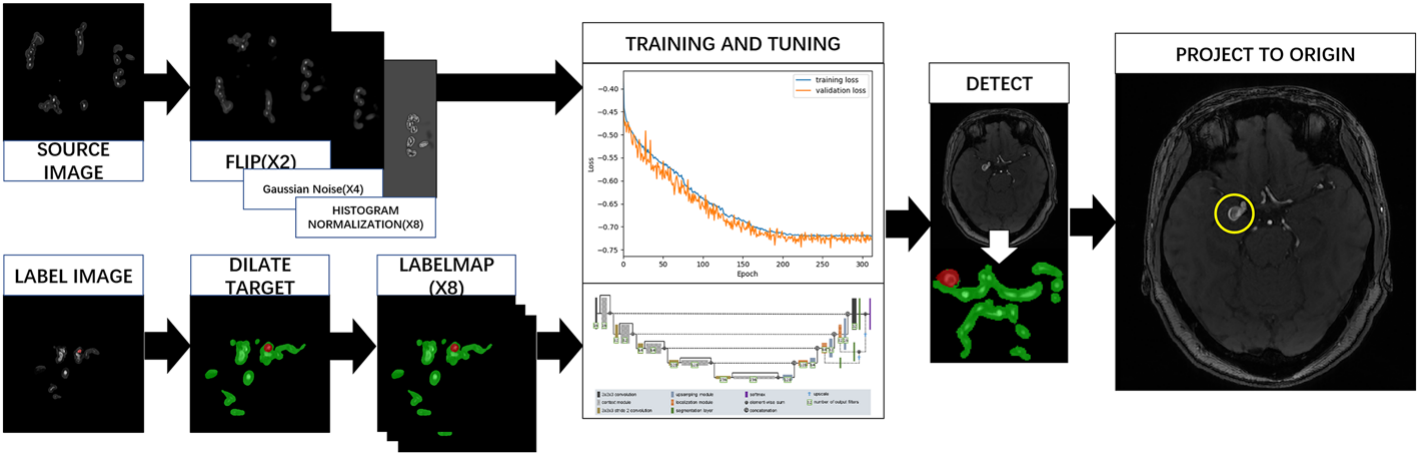
Workflow of the aneurysm detection using 3D-UNET

After training, we obtained the deep neural network model and used it to predict the new TOF-MRA image; the image was processed by step one, got the vessel area, then the model would predict each voxel in the vessel area. The model would give the likelihood of each voxel to be normal vessel or aneurysm. These possibilities were binarized at a threshold of 0.5, a value greater than 0.5 was converted to 1 and a value less than 0.5 was converted to 0. Then each voxel of the vessel area was classified into 2 labels: vessel area and aneurysm area. The voxels of one label were all connected to be one component, so we took the center of the aneurysm area and draw a sphere from this point, with the radius the same as used to dilate the annotations. The area inside the sphere had a high probability to be an aneurysm, and clinicians could check the area more carefully to make the diagnosis (Fig. 6).

## Data Availability

Not applicable.

## Abbreviations

TOF-MRA: Time-of-flight magnetic resonance angiography
DSA: Digital subtraction angiography
CNN: Convolutional neural networks
FCN: Fully convolutional network
CAD: Computer-assisted detection
CPU: Computer-assisted detection
RAM: Random access memory
GPU: Graphic processing unit
MCA: Middle cerebral artery
PCA: Posterior cerebral artery
ICA: Internal carotid artery
ACA: Anterior cerebral artery
BA: Basilar artery
VA: Vertebral artery
FP: False-positive cases
DICOM: Digital Imaging and Communications in Medicine (DICOM) is the standard for the communication and management of medical imaging information and related data
